# A Premature Stop Codon in RAF1 Is the Priority Candidate Causative Mutation of the Inherited Chicken *Wingless-2* Developmental Syndrome

**DOI:** 10.3390/genes10050353

**Published:** 2019-05-09

**Authors:** Ingrid Youngworth, Mary E. Delany

**Affiliations:** Department of Animal Science, University of California, Davis, Davis, CA 95616, USA; iyoungworth@ucdavis.edu

**Keywords:** development, chicken, RASopathy, capture array, limb development

## Abstract

The chicken *wingless*-2 (*wg-2*) mutation is inherited in an autosomal recessive fashion, and the resulting phenotype in mutant (*wg-2*/*wg-2*) individuals is a developmental syndrome characterized by absent wings, truncated legs, craniofacial as well as skin and feather defects, and kidney malformations. Mapping and genotyping established that the mutation resides within 227 kilobases (kb) of chromosome 12 in a *wg-2* congenic inbred line. A capture array was designed to target and sequence the candidate region along with flanking DNA in 24 birds from the line. Many point mutations and insertions or deletions were identified, and analysis of the linked variants indicated a point mutation predicted to cause a premature stop codon in the *RAF1* gene. Expression studies were conducted inclusive of all genes in the candidate region. Interestingly, *RAF1* transcription was elevated, yet the protein was absent in the mutants relative to normal individuals. *RAF1* encodes a protein integral to the Ras/Raf/MAPK signaling pathway controlling cellular proliferation, and notably, human RASopathies are developmental syndromes caused by germline mutations in genes of this pathway. Our work indicates *RAF1* as the priority candidate causative gene for *wg-2* and provides a new animal model to study an important signaling pathway implicated in limb development, as well as RASopathies.

## 1. Introduction

**Figure 1:**
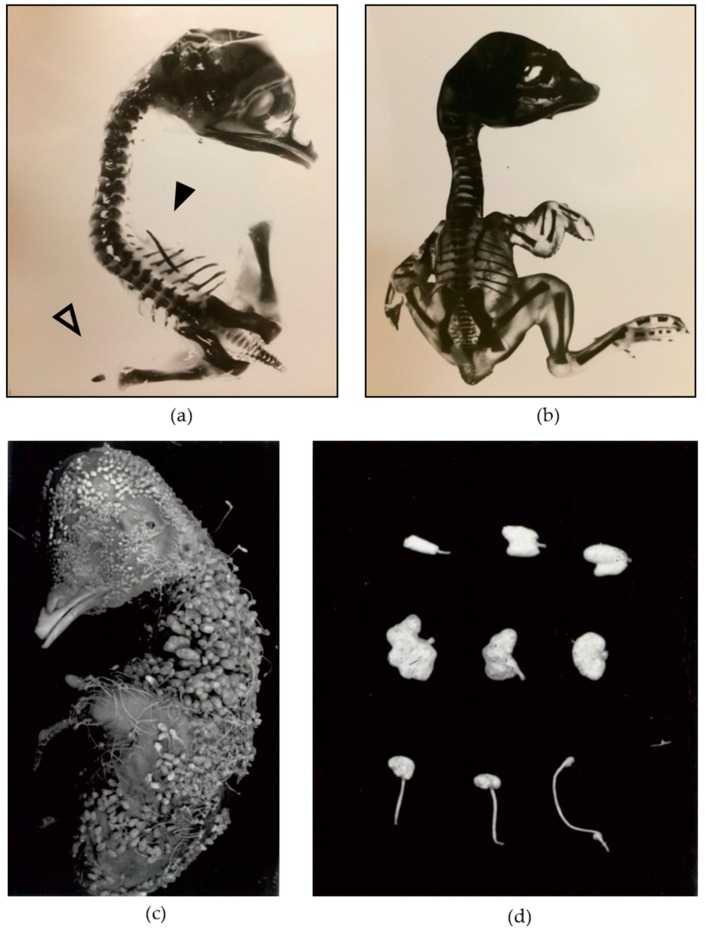
**Phenotype of the *wg-2* mutant illustrating wing, leg, and feather abnormalities.** Shown here is (**a**) a *wg-2* mutant embryo missing all elements of the forelimbs (wings) (solid arrowhead) and exhibiting truncated hindlimbs (legs) (open arrowhead), as compared with (**b**) a normal, wild-type embryo with limbs on the right side extended for visibility. The embryos were incubated to 18 days of incubation (DI) (mutant) and 19 DI (normal) and stained with Alizarin Red to highlight bone structure. (**c**) An 18 DI *wg-2* embryo illustrates the severe feather abnormality. (**d**) Several *wg-2* feathers are shown to portray the range of the abnormality. The top two rows show the most severely affected feathers: large and abnormally compound feathers lacking a filamentous shaft. The bottom row illustrates the more normal filamentous feathers that have basal dilations (called clubbed down and observed in other lines) and sometimes “blebs” at the distal tip of the filament (associated with the *wg-2* phenotype). Reproduced and adapted with permission from Jacqueline Pisenti, “Genetic and environmental influences on the expression of the mutation *wingless-2* in the chick embryo;” published by the University of California, Davis [7].

The chicken has long been employed as a research model to investigate vertebrate development, with many contributions to the understanding of limb formation [1]. The *wingless-2 (wg-2)* mutation is one of several inherited defects identified in chicken and offers an opportunity as a unique model to discover genes and pathways previously unknown to be involved in development. The phenotype of affected homozygous mutants is characterized by absent forelimbs, truncated hindlimbs, craniofacial malformations such as clefting or cross beak, and kidney and severe skin/feather malformations (Figure 1a,c,d). Early work employing tissue recombination techniques established that the ectoderm was likely the defective tissue layer in the mutant limb buds [2], although the mesoderm loses the ability to support wing growth at stages soon after the ectoderm defect is noted [2,3].

The *wg-2* mutation is autosomal recessive and embryonic lethal [3]; thus, phenotypically normal heterozygous carriers are viable. The *wg-2* congenic inbred line (Wingless-2.331) was created by repeatedly crossing *wg-2* carriers into the highly-inbred UCD (University of California, Davis) 331 line, selecting carrier parents each generation (confirmed by test mating) before the resulting birds were crossed inter se and the new line was closed [4,5]. The uniform genetic background conferred by UCD 331 (>99% inbred) [6] facilitated the use of genetic and genomic techniques such as SNP arrays, linkage mapping, and the capture array described herein to study the *wg-2* introgressed candidate region.

The DNA retaining the causative element (also known as the causative region) was first mapped to a 2-Mb span of chromosome 12 (GGA 12) by employing a 3K SNP array; the region’s size was successively narrowed over several generations by genotyping birds at linked SNPs [5]. These techniques ultimately mapped the causative element to a 227-kb region on GGA 12 [5]. A prior study testing the capture array technique in chicken used two pooled mutant individuals and identified a large amount of sequence variation in the causative region in the *wg-2* line [8].

**Figure 2:**
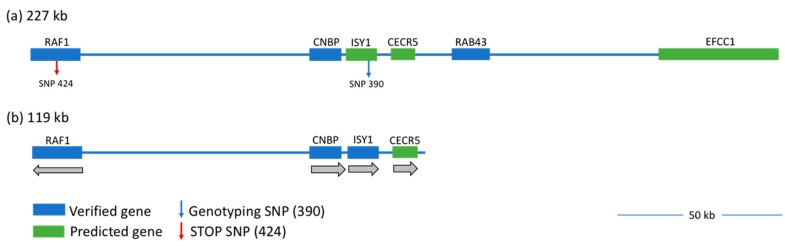
**The *wg-2* linked region of chromosome 12 is shown at different stages of genomic analysis.** The candidate region initially spanned 227 kb (**a**), from chr12:4,958,991–5,185,627 on build galGal5 and contained six genes: three verified (blue) and three predicted (green). The diagnostic SNP 390 is indicated (blue arrow to the right within *ISY1*) and is identifiable in NCBI dbSNP as rs14034687. The premature STOP SNP in *RAF1* (red arrow to the left) is identified as SNP 424 (rs314452077). The newly-identified and smaller linked region of 119 kb is indicated in (**b**) with genes eliminated from the 3′ end (*EFCC1*, *RAB43*) and additional intergenic DNA no longer linked to *wg-2* (new 3′ endpoint: 5,078,217). *CNBP*, the predicted gene *CECR5*, and part of *RAF1* (14,462 nt of 27,936 total) remain in the candidate region, as well as *ISY1*, updated as a validated gene here (blue), as our results showed *ISY1* transcripts present in chicken embryos. Grey arrows below each gene (in (**b**)) indicate the direction of transcription along the strand. Note that as *RAF1* is transcribed on the opposite strand, the 5′ end of the transcript is retained in this linked region. A 50-kb bar is included for scale.

The region of interest, 227 kb retaining the causative mutation, spans from 4,958,8991–5,185,627 on GGA 12 in the Gallus_gallus-5.0 (galGal5) assembly (Figure 2a). As per the UCSC Genome Browser [9], this region contains three RefSeq genes (*RAF1*, *CNBP*, also known as *ZCCHC13*, and *RAB43*), as well as three additional Ensembl-predicted protein-coding genes (*ISY1*, *CECR5(L)*, also known as *HDHD5*, and *EFCC*, also known as *CCDC48*) [5]. This region is syntenic in the genomes of several species, such as human, mouse, and other vertebrates, and so, the genes in those organisms are predicted in chicken, although not yet annotated. *RAF1* (also known as *RAF* proto-oncogene serine/threonine-protein kinase) is a regulator of cell migration, proliferation, and differentiation [10,11,12]. *RAF1* is known as a regulator of the Ras/Raf/MAPK pathway, which is also implicated in human cancer via these processes, as well as cell cycle regulation and angiogenesis [10,13]. *CNBP* (CCHC-type zinc finger nucleic acid binding protein) is also involved in cell proliferation [14] and differentiation [15] and is both a positive and negative transcriptional regulator [16,17]. *RAB43* (Ras-related protein RAB43), although less understood, is part of the GTPase superfamily and has a role in the maintenance of the Golgi complex and ER-Golgi trafficking [18,19]. *ISY1* (pre-mRNA-splicing factor *ISY1* homolog) is known only to have a function in the splicing complex in *Saccharomyces cerevisiae* [20,21]. Little is known about *CECR5*/*HDHD5* (cat eye syndrome chromosome region, candidate 5), but it is indicated as a candidate gene for a human developmental disorder called cat eye syndrome [22]. Finally, and similarly, *EFCC1*/*CCDC48* (EF-hand and coiled-coil domain containing 1) is not well-studied, but is predicted to be involved in calcium ion binding [23].

The ultimate aim of our research is to determine the causative sequence element responsible for the *wg-2* phenotype; to this end, our analysis identified a single-nucleotide variant (SNV) that was predicted to result in a premature stop codon in the *RAF1* gene transcript. Further work examined the expression of *RAF1* and the other genes in the linked region in mutants, carriers, and wild-type birds via RT-qPCR and Western blotting. Our results support the hypothesis that this SNV causing a premature stop codon in *RAF1* is a high priority candidate as the causative mutation for the *wg-2* phenotype. Errors in *RAF1* or other members of the Ras/MAPK pathway are termed RASopathies in humans and are phenotypically similar to *wg-2* in that multiple developmental systems are affected, and some clinical features of these disorders are shared [24]. Though RASopathies do not typically include limb abnormalities [24], the Ras/MAPK pathway has also been shown to be involved in limb development specifically [25].

## 2. Materials and Methods

### 2.1. Genetic Lines

All samples studied were from the UCD Wingless-2.331 developmental congenic inbred chicken line. The line was established at UCD by breeding carriers from the originating population maintained at the University of Connecticut, Storrs [4]. Birds were genotyped at a tightly-linked SNP (SNP 390) and test mated to identify heterozygous carriers, which were bred inter se to maintain the line. Wild-type birds and carriers were phenotypically normal and were designated as +/+ and +/*wg-2* respectively, and the mutant individuals as *wg-2/wg-2*. The genetic line was bred and maintained according to the procedures in an approved animal care protocol (Protocol #18816).

### 2.2. Capture Array and Next-Generation Sequencing 

#### 2.2.1. Sample Collection

Eggs collected from +/*wg-2* by +/*wg-2* (carrier) matings were incubated at 37 °C and 50% humidity for 11 days to reach Stage 36 HH (Hamburger and Hamilton) [26]. This is the equivalent to 10 days of incubation (DI) of non-inbred lines, as the congenic inbred line individuals are typically delayed ca. 24 h in their developmental progression. Embryos were removed from the eggs and preserved in RNAlater at −20 °C. The extraembryonic membranes of each embryo were sampled and used to isolate DNA via the DNeasy Blood and Tissue Kit (Qiagen; Hilden, Germany). This DNA was used to determine or verify the *wg-2* status of the embryos by genotyping via TaqMan qPCR at a tightly-linked SNP (SNP 390) [5]. Two +/+ and 18 *wg-2/wg-2* embryos were collected in this manner. Blood was drawn from 4 +/*wg-2* parents, and DNA was isolated and genotyped using the same process. The set of 24 individuals was used to improve resolving power to distinguish the potentially causative mutations from normal inter-individual variation within the line.

#### 2.2.2. Capture Array and Sequencing

Isolated genomic DNA was sent to the QB3 Vincent J. Coates Genomics Sequencing Laboratory (University of California, Berkeley) to capture and sequence the selected genomic region. This region included the 227-kb linked region of GGA 12, as well as flanking DNA for a total of 300 kb. Wafergen/Takara PrepX kits were used for library preparation of the 24 genomic DNA samples, and target enrichment was performed with the SeqCap EZ Choice enrichment system and NimbleGen SeqCap EZ probes (Roche; Pleasanton, WI, USA). Sequencing was done on one lane of a MiSeq with v2 kit on 150 cycles with paired-end reads (Illumina; Hayward, CA, USA). The reads are available in the NCBI Sequence Read Archive, under BioProject Accession PRJNA531717.

#### 2.2.3. Bioinformatic Analysis

Illumina reads were trimmed and analyzed for quality with TrimmomaticPE (v0.36) [27]. Alignment to Gallus_gallus-5.0 (galGal5) was accomplished using Bowtie2 (v2.2.9) [28]. Samtools was used for file conversions and pileup file creation (v1.3.1) [29] and Picard for duplicate marking (v2.8.0) [30]. VarScan was used to call variants (v2.4.2) [31], and SnpEff (v3.6) [32] and Ensembl’s Variant Effect Predictor [33] were used to predict the variant effects.

### 2.3. RT-qPCR

#### 2.3.1. Sample Collection

Samples were collected as in the previous section, but eggs were incubated only to 6 DI to obtain Stage 26 HH embryos [26]. The status of the RAF1 SNP (rs314452077, referred to as SNP 424) was examined in addition to SNP 390, the diagnostic SNP for *wg-2*. At Stage 26 HH, the wings and legs were clearly present [26]. While mutants did have early limb buds that appeared similar to those of wild-type embryos at several stages prior to Stage 26 HH, they are very clearly distinguishable from normal limbs by this point.

#### 2.3.2. RNA Isolation

Whole embryo samples were homogenized, and RNA was isolated using the PureLink RNA mini kit (Invitrogen; Carlsbad, CA, USA) using the protocols provided. DNase treatment to remove DNA from the RNA samples was performed, briefly: DNase I was added to the sample, incubated for 10 min, and DNase Inactivation Reagent was added to stop the reaction (RNAqueous-4PCR, Ambion; Austin, TX, USA). A NanoDrop 2000 spectrophotometer (Thermo Fisher Scientific; Wilmington, DE, USA) was used for quantification of the samples and general quality assessment.

#### 2.3.3. cDNA Reverse Transcription

Using total RNA from the previous section, 10 μL of RNA were added to 10 μL of Master Mix from the High Capacity cDNA Reverse Transcription Kit (Applied Biosystems; Vilnius, Lithuania). Reactions were placed in a Benchmark Scientific TC9639 Thermal Cycler and run on a program of 25 °C for 10 min, 37 °C for 120 min, and 85 °C for 5 min. The resulting cDNA product was stored briefly at 4 °C and long term at −20 °C.

#### 2.3.4. TaqMan Gene Expression Assays

Using total cDNA from the previous section, six samples (two per genotype) were examined in triplicate wells on a 96-well plate to compare transcript levels for a gene of interest to *GAPDH* transcript expression. A TaqMan assay for each of the six genes of interest (Table 1) was performed on its own plate. In each well, 2 μL of cDNA were added to a mix of 10 μL TaqMan Fast Advanced Master Mix (2×) (Applied Biosystems; Austin, TX, USA), 1 μL custom TaqMan Assay (20×) (Applied Biosystems; Pleasanton, CA, USA), and 7 μL of nuclease-free water. Each plate was placed in a Benchmark Scientific TC9639 Thermal Cycler and run on a program of 2 min at 50 °C, 20 s at 95 °C, and then 40 cycles of 1 s at 95 °C and 20 s at 60 °C.

#### 2.3.5. Quantitative Analysis

Expression analysis was performed via the ddCt method [34] with adjustment for less than 100% PCR efficiency. Unpaired two-tailed *t*-tests were performed to determine the significance of the results, and Bonferroni correction was used to correct for multiple comparisons [35].

### 2.4. Western blotting

#### 2.4.1. Sample Collection

Samples were collected as in Section 2.2.1, but 11 DI dissected embryos were immediately flash-frozen in liquid nitrogen and stored at −80 °C. Four samples of each genotype were used in this experiment (*n* = 12 embryos).

#### 2.4.2. Protein Extraction

Frozen embryos were ground by a motor and pestle and cells lysed by vortexing and the addition of Tissue Protein Extraction Buffer (T-PER, Thermo Fisher Scientific; Rockford, IL, USA). The supernatant containing the protein was removed after centrifugation, and aliquots were mixed 1:1 with 2× Laemmli buffer with 2-mercaptoethanol and boiled for 10 min at 100 °C to denature the proteins. Denatured protein samples were stored at −20 °C after quantification and quality assessment by a NanoDrop 2000 Spectrophotometer (Thermo Fisher Scientific; Wilmington, DE, USA).

#### 2.4.3. SDS-PAGE and Protein Transfer

Precast 4–15% Mini-PROTEAN TGX 10-well gels (Bio-Rad; Hercules, CA, USA) were run at 100 V for 90 min in the Mini-PROTEAN Tetra Cell (Bio-Rad; Hercules, CA, USA) to separate proteins in the samples, and the separated proteins were transferred to polyvinylidene fluoride (PVDF, Bio-Rad; Hercules, CA, USA) membranes by use of a cassette in the same chamber powered at 100 V for 1 h.

#### 2.4.4. Western Blotting

The PVDF membrane with transferred proteins was briefly stained with Ponceau S to observe successful protein transfer and destained with water before proceeding to antibody incubations. The membrane was blocked overnight in OneBlock Western CL blocking solution (Genesee Scientific; San Diego, CA, USA) at 4 °C prior to incubation in primary antibody for 1–2 h at room temperature on a rocker. Two brief rinses and three 5-min washes with Tris-buffered saline with Tween 20 (TBST) were performed, and the membrane was then incubated in secondary antibody for 1 h at room temperature on a rocker. The washes were repeated, with the final wash replaced by Tris-buffered saline (without Tween, TBS). The membrane was incubated in Luminata Forte HRP Chemiluminescence developer (Millipore Sigma) for two minutes prior to visualization on the ChemiDoc-ItTS2 Imager (UVP; Upland, CA, USA). All antibodies were diluted in OneBlock Western CL (Genesee Scientific; San Diego, CA, USA). Primary antibodies were from rabbit and developed to react to human sera, and secondary antibody was goat anti-rabbit conjugated to horseradish peroxidase (Table 2). Secondary-only controls were run for each of the three protein targets to ensure specificity; no bands appeared on these controls. Each sample for each protein was blotted in triplicate.

## 3. Results

### 3.1. Capture Array and Next Generation Sequencing

In the 227-kb linked region of DNA captured and sequenced from the 24 samples, 1,937 SNVs and 298 indels were called. These variants were filtered to select only those that could be potentially causative according to the following criteria: causative variant(s) must be homozygous in mutants, heterozygous in carriers, and homozygous for the alternate allele in wild-type. This left 503 SNVs and 53 indels as potentially causative in the 227-kb linked region (Table 3). The size of the region delimited by these variants was 119 kb (Figure 2b); thus, we achieved a significant reduction in size of the causative region (by >100 kb) that remained linked to *wg-2* and encoded the causative mutation. Ensembl Variant Effect Predictor (VEP) and SnpEff were used to predict the effects of these variants and sort them into categories: non-coding (occurring in an intron, 5000 bases upstream or downstream of a gene, intergenic, or in a non-coding transcript) and coding (in a predicted splice region, 3 prime UTR, or exon). Generally, non-coding variants were defined as having minimal or low consequences and coding as severe, high, or moderate by these tools.

All indels identified were predicted to have minimal consequences: occurring in an intron, up- or down-stream of a gene, or in a non-coding transcript. The vast majority of the SNVs also fell into these categories. Just four SNVs appeared in predicted splice regions (within 1–3 bases of the exon or 3–8 bases of the intron), and nine appeared in exons. Even for these exonic variants, most were unlikely to have a strong effect: eight resulted in synonymous mutations. The single exception was an SNV that caused a cytosine to thymine transition, resulting in a predicted stop codon in the transcript for RAF1 in *wg-2/wg-2* individuals. The position of this variant was toward the end of exon 2, at base 4,965,864 on galGal5 (rs314452077). Querying the four predicted splice region variants and this exonic SNV in dbSNP via the UCSC Genome Browser [9] showed that the splice variants had all been observed by other researchers in different chicken populations; only the exonic SNV had not been observed elsewhere. As it was the only unique variant linked to *wg-2* with a serious predicted physiological consequence, we identified it as the priority candidate causative mutation for further investigation.

### 3.2. RT-qPCR

The ddCt method of relative quantitative transcript analysis was employed for those genes that showed expression in the Stage 26 HH embryos: *RAF1*, *CNBP*, and *ISY1*. Transcription of the other genes in the region, *RAB43*, *EFCC1*/*CCDC48*, and *CECR5*/*HDHD5*, was not detected by RT-qPCR in any of the samples. *GAPDH* was expressed normally on these plates, but sample wells for these three genes (*RAB43*, *EFCC1*/*CCDC48*, and *CECR5*/*HDHD5*) were not distinguishable from the no-template controls even after 40 cycles. Of the three expressed genes (*RAF1*, *CNBP*, and *ISY1*), only *RAF1* produced results that were found to be statistically significantly different on a genotype basis. The *RAF1* expression pattern indicated step-wise genotype differences among +/+, +/*wg-2*, and *wg-2/wg-2* individuals (Table 4), with the lowest expression in wild-type and the highest in mutants. Mutant (*wg-2/wg-2*) individuals had a 1.7-fold higher expression than that in wild-type (+/+) (*p* = 0.001) and 1.2-fold higher than in carriers (+/*wg-2*); carriers indicated 1.4-fold higher levels than wild-type.

### 3.3. Western blotting

**Figure 3:**
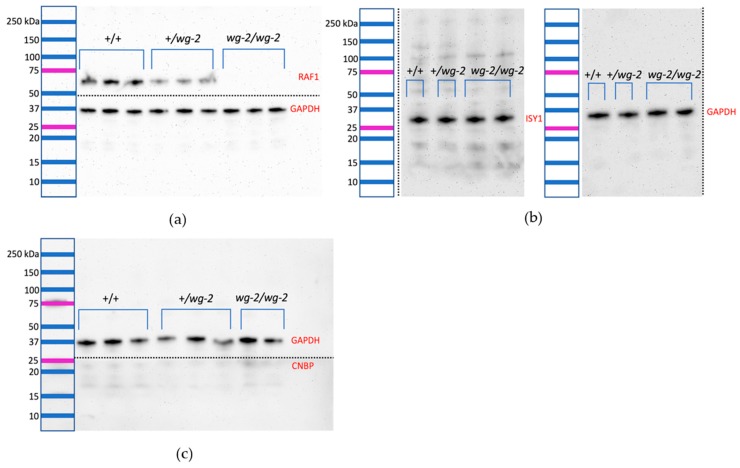
Western blots of RNA-expressed genes (*RAF1*, *CNBP*, *ISY1*) to detect protein in embryos (Stage 36 HH) of the three genotypes of the Wingless-2.331 line show that *wg-2/wg-2* mutant embryos lack RAF1 protein expression. Precision Plus Protein Dual Color standards (Bio-Rad; Hercules, CA, USA) are shown at the left to indicate the approximate size of the bands. Dotted lines indicate cuts made in the PVDF membrane in order to incubate pieces separately using different primary antibodies. In total, *n* = 12 individuals (four each of: wild-type, carrier, and mutant) were examined for RAF1, ISY1, and CNBP protein expression as compared to GAPDH. Three technical replicates for each individual were performed. (**a**) shows a representative image of two biological replicates of each genotype with bands for RAF1 (above the dotted line, 73 kDa) and GAPDH (below the dotted line, 35 kDa). The average ratio of RAF1 expression compared to GAPDH in wild-type in this blot was 2.08, whereas this ratio was 0.91 in carriers and zero in mutants (calculated using ImageJ software, Version 1.51). This supports the visual assessment that carriers have roughly half the RAF1 expression of wild-type individuals. (**b**) shows a representative image of a wild-type, carrier, and two mutant individuals with bands for ISY1 (left blot, 33 kDa) and GAPDH (right blot, 35 kDa). Expression appears equivalent across samples, and ImageJ for this blot was used to calculate an ISY1/GAPDH ratio of 0.95 for wild-type, 1.13 for carrier, and 1.00 for the average of the two mutants. (**c**) shows a representative image of three wild-type, three carrier, and two mutant individuals for CNBP (bottom, 19 kDa) and GAPDH (top, 35 kDa). A strong band did not appear for CNBP even at high concentrations of primary antibody, and the faint band at the predicted weight (19 kDa) did not register above the background in most lanes, as analyzed by ImageJ.

Those genes in the region with detectable transcripts were examined for their protein expression (RAF1, CNBP, and ISY1). RAF1 exhibited a distinct band at its predicted weight of 73 kDa both in +/+ and +/*wg-2,* but was absent in *wg-2/wg-2* samples (Figure 3a). Though present, this band appeared weaker in +/*wg-2* samples (Figure 3a, Table 4). ImageJ quantitative analysis supported the qualitative step-wise expression: for the blot shown (Figure 3a), the ratio of the RAF1 band intensity to GAPDH averaged 2.08 in wild-type samples as compared to 0.91 in carriers, and as noted, there was no RAF1 band in mutants. Blotting was performed in triplicate repetition of each sample, and the average expression ratio of RAF1/GAPDH was 2.26 in wild-type, 0.87 in carriers, and zero in mutants. Expression of GAPDH (35 kDa) was observed for every individual and genotype and was always present for each sample on the same membrane (this was consistent for ISY1 and CNBP as well).

ISY1 exhibited a distinct band at its predicted weight of 33 kDa, as well as additional faint bands at higher molecular weights (Figure 3b). There was no visible difference among samples of different *wg-2* genotypes for ISY1, indicating that there was likely not a significantly different level of protein in *wg-2/wg-2* birds as compared to +/+ or +/*wg-2*. Consistent results were obtained for all samples for each genotype and for each replicate per sample. ImageJ quantification for this blot supported the visual observation, where the ratio of ISY1 band intensity to GAPDH was 0.95 in wild-type, 1.13 for carrier, and 1.00 average for the two mutants. The average expression for the triplicate observations for each sample indicated an ISY1/GAPDH ratio of 1.02 in wild-type, 1.12 in carriers, and 1.14 in mutants. Thus, a subtle increase in protein expression was indicated in carriers and mutants compared to the wild-type.

CNBP blots exhibited very faint bands appearing in each lane at approximately the correct weight (19 kDa) along with smaller and larger bands. Lanes for different genotypes looked roughly equivalent (Figure 3c). Although ImageJ was used to attempt to quantify the CNBP signal present, the bands did not register above the background, suggesting no detectable expression at the resolution level of Western blotting.

## 4. Discussion

The deep sequencing of the linked candidate region for *wg-2* in a congenic inbred line identified a large number of variants in the region. In all, we found 1,937 single-nucleotide variants or point mutations (also known as SNVs) and 298 insertions or deletions (also known as indels) in the 227-kb linked region of chromosome 12 in the *wg-2* line as compared to the reference sequence. However, a potentially causative mutation in this case must be homozygous for one allele in all affected (i.e., mutant) individuals, heterozygous in all carriers, and homozygous for the alternate allele in wild-type birds. Of the total 2235 variants found, 503 SNVs and 53 indels met these criteria. Our use of currently-available prediction tools expedited the analysis of large numbers of variants and informed further consideration of how the variant itself could have a meaningful effect on a phenotype. A single exonic variant was the only one predicted to have a high impact coding effect by creating a nonsense mutation in two transcripts of a gene, as per VEP and SnpEff output. This variant is a SNV near the end of exon 2 (a cytosine to thymine transition on the reverse strand at base 4,965,864 on GGA12 on build galGal5) in the *RAF1* gene that generates a premature stop codon (UGA), which if translated would result in a severely truncated RAF1 protein. This mutation was also observed in the initial capture array performed using two individuals from the line and was submitted to NCBI dbSNP (rs314452077) [8]. For clarity, this variant will be referred to as SNP 424 in reference to its original submission to the database (DELANY|WG2-SNP-424).

The chicken *RAF1* gene is 27,938 bp long (4,945,518-4,973,456 bp on the reverse strand of chromosome 12, galGal5), with three splice variants identified (ENSGALG00000004998) [36]. The canonical isoform as described by UniProt generated a protein of 647 amino acids and 73 kDa [37] encoded by an mRNA of 2,182 bases and 17 exons [38]. As *RAF1* errors are known to be causative of developmental defects in humans and no other sequence changes were predicted to have a likely effect on expression, we proposed this mutation as the most likely cause of the *wg-2* phenotype. We examined the hypothesis that the premature stop codon in *RAF1* in *wg-2/wg-2* mutants was the causative element for the *wg-2* phenotype by studying both RNA transcript and protein expression. However, variants specific to *wg-2/wg-2* could potentially affect the expression of other genes in the region even in the absence of obvious predicted effects. Thus, to be as unbiased as possible, we also assessed transcript (Table 4) expression of the other five known and predicted genes in the candidate region (*CNBP*, *ISY1*, *RAB43*, *CECR5*, and *EFCC1*) and subsequently examined protein expression for the genes with detectable transcripts (Figure 3; *CNBP, ISY1*, and *RAF1*). In doing so, we are recognizing that the causation of an inherited phenotype could also be due to effects from altered splicing or non-coding elements.

In the transcript studies, whole embryos were examined, such that limb buds were clearly present, although not fully developed (Stage 26 HH, ca. 6 DI) [26], to study relevant (in terms of the phenotype) early developmental expression. Of the six genes examined, *RAF1*, *CNBP*, and *ISY1* had measurable mRNA expression, whereas *RAB43*, *EFCC1*/*CCDC48*, and *CECR5*/*HDHD5* transcripts were not detected. Interestingly, the *RAF1* RNA expression levels were significantly higher in mutants than in carriers and homozygous normal individuals. Thus, the nonsense mediated decay (NMD) pathway, known for its canonical role in impacting abnormal transcripts [39,40,41], did not appear to be operating in suppressing *RAF1* expression. This may be due to the position of the premature stop codon, which is close to the next natural splice junction [42]. Rather, we found elevated levels of transcript in the mutants that could be produced in response to an insufficient functional protein, especially as *RAF1* is normally ubiquitously expressed [10,43]. In comparison, *CNBP* and *ISY1* did not have significantly different RNA expression between the mutant individuals and the carriers or wild-type.

Those genes with expressed transcripts (*RAF1*, *CNBP*, and *ISY1*) were then examined at the protein level by Western blotting in additional Stage 26 HH embryos. CNBP in particular indicated limited protein expressed at this stage, with very faint multiple bands of identical intensity and pattern among the three genotypes. ISY1 had a clear band at its predicted weight, along with several fainter bands at higher weights, and carriers and mutants had subtly higher expression than wild-type individuals. Though *ISY1* is not yet listed as a RefSeq gene in the UCSC annotation track for chicken [9], our expression results support that *ISY1* is a real gene. RAF1 exhibited a strong band at its predicted weight (73 kDa) in wild-type individuals and carriers but was absent in mutants (Figure 3a). The carrier birds had a visibly lighter band as compared to wild-type, indicating that the one functional copy of the *RAF1* gene was not able to produce +/+ protein levels, i.e., there did not appear to be dosage compensation at the protein level. However, as carriers are phenotypically normal, this suggested a single functional copy of the gene was sufficient.

*RAF1* (also known as Raf-1 proto-oncogene) is a serine/threonine kinase and a component of the Ras/Raf/mitogen-activated protein kinase (MAPK) pathway [37]. Mutations in the components of this pathway, frequently missense mutations that result in hyperactivation of the pathway, cause developmental syndromes with a variety of overlapping features termed RASopathies [24,44,45]. Receptor tyrosine kinases, crucially including FGF receptors, start the signaling cascade by activating Ras, which recruits Raf genes in order to activate MEK1/2, which in turn activate ERK1/2 (also known as MAPK) [10,12,46,47]. Though the overall activity of this pathway was not assessed in *wg-2*, it is likely that absent RAF1 results in overall loss of function, unlike a typical RASopathy. FGF activity, which of course is crucial to many aspects of development, is frequently mediated by ERK1/2 [48]. Staining of phosphorylated (i.e., activated) ERK expression in mouse embryos indicated a close association with limb bud outgrowth, as well as the frontonasal process and several other regions [25]. Further, specific interactions of FGF with MAPK play a part in chick limb elongation via gradient-directed cell motility [49]. Manipulations of Pyst1/MKP3 in chicken, an antagonist of ERK1/2, show it to be regulated by FGF signaling in chick limb buds and capable of truncating limb outgrowth when overexpressed [50]. Given this and further evidence of Ras/MAPK pathway involvement in limb development, it is surprising that RASopathies do not typically include limb abnormalities.

*RAF1* mutations in humans have been implicated in two specific RASopathies, Noonan syndrome and Noonan syndrome with multiple lentigines (NSML, formerly known as LEOPARD syndrome), both of which are characterized by dysmorphic craniofacial features, congenital heart defects, and short stature, among other clinical features [44]. Interestingly, NSML exhibits a skin malformation as does *wg-2* (lentigines in NSML and feather dysmorphia in *wg-2*; see feather abnormalities in Figure 1c,d), with all three syndromes presenting craniofacial abnormalities [7,44]. Though they are less consistent features, *wg-2* mutants can sometimes display incomplete eyelids, tail abnormalities, and heart defects [51]. Differences in overlapping function of the Raf genes between chicken and human may explain some of the phenotype variation, as chicken has only two, *RAF1* (also known as *CRAF*) and *BRAF*, whereas human has three Raf genes: *ARAF*, *BRAF*, and *RAF1*. All three Raf genes are also present in mice, but mouse knockouts of *Craf-1* (the *RAF1* equivalent) are lethal as in chicken, exhibiting skin (thinner and poorly-differentiated dermal and epidermal layers, small and underdeveloped hair follicles) and lung defects, as well as overall growth retardation, sometimes abnormally-fused eyelids and reduced livers (variation was observed depending on the background strain) [52,53,54]. Strikingly, when mice are missing three copies of the total four between Braf and Craf-1, lethality is earlier in development and the additional phenotypic abnormalities include underdevelopment of limbs [55]. This sensitivity to Raf dosage presents an opportunity to explore the respective roles and redundancies of the different Raf genes in mammals, especially as compared to their chicken orthologs. It is noteworthy that RASopathies are not typically described as lethal, and we speculate that a complete knockout of *RAF1*, as in *wg-2* and mouse models, could not be tolerated in humans. A plausible hypothesis is that complete absence of chicken RAF1 results in overall loss of function of the pathway, unlike a typical RASopathy. An assessment of the overall activity of the pathway would test this hypothesis. Notably, however, *RAF1* is involved in developmental processes independent of its canonical activation of MEK1/2 in the MAPK pathway [12,56]. It is unknown to what extent the *wg-2* phenotype is caused by the dysregulated MAPK pathway versus these other processes, and so, this model presents a novel opportunity to explore the expanded role of *RAF1* in limb and other development in these different pathways. Elucidating the involvement of *RAF1* in chicken limb development will inform about the vertebrate Raf gene family and understanding of their overlapping functions.

## 5. Conclusions

Many years of research studying *wg-2* succeeded in detailed characterization of the phenotype, creation of a congenic inbred line, and mapping the mutation to an increasingly smaller candidate region on GGA 12 with elimination of candidate genes and potentially causative variants. Finally, a single variant was presented as a high-probability candidate for causation of the phenotype. Future generations of *wg-2* birds will be genotyped at both SNP 390 and SNP 424 alongside testing mating in carriers to assess recombination events and further resolve the causative region. In terms of the next steps in research, establishment of the *RAF1* premature stop codon in *wg-2* as the definitive causative mutation would benefit from in vivo research, demonstrating that the presence of exogenously-derived wild-type/functional RAF1 in *wg-2* mutants rescues the phenotype and/or recreating the mutation (via knockouts using CRISPR) and finding that absence of the protein recapitulates the mutant phenotype in wild-type birds. Nonetheless, the *wg-2* chicken model continues to contribute to understanding of avian and vertebrate development of limbs.

## Figures and Tables

**Table 1:** TaqMan assays were used to investigate transcript presence and quantity from genes identified within the *wg-2* 227-kb GGA 12 linked region.

Gene Target	Assay ID	Genome Build	Amplicon Size (Bases)	Dye
*RAF1*	Gg03349021_m1	galGal2	127	FAM-MGB ^1^
*CNBP*	Gg03339297_m1	galGal2	114	FAM-MGB ^1^
*ISY1*	Gg03328872_m1	galGal2	71	FAM-MGB ^1^
*RAB43*	Hs03006628_gH	GRCh38 ^2^	59	FAM-MGB ^1^
*CECR5*	Hs00215190_m1	GRCh38 ^2^	68	FAM-MGB ^1^
*EFCC1*	Hs01088833_m1	GRCh38 ^2^	99	FAM-MGB ^1^

^1^ FAM-MGB is 6-carboxyfluorescein dye attached to a minor groove binder probe. ^2^ TaqMan assays for *RAB43*, *CECR5*, and *EFCC1* were designed based on human genome sequences rather than chicken, as chicken assays were not available.

**Table 2:** Details for the primary and secondary antibodies used for Western blotting to detect protein expression among *wg-2* genotypes from expressed genes of the 227-kb candidate region on GGA 12.

Protein Target ^1^	Company	ID	Host Species ^1^	Clonality	Dilution
RAF1	Abcam	ab181115	Rabbit	monoclonal	1:1000
CNBP	Abcam	ab83038	Rabbit	polyclonal	1:150
ISY1	Novus Biologicals	NBP1-81865	Rabbit	polyclonal	1:250
GAPDH ^2^	Novus Biologicals	NB300-322	Rabbit	polyclonal	1:2000
Rabbit IgG ^3^	Abcam	ab205718	Goat	polyclonal	1:50,000

^1^ All primary antibodies for RAF1, CNBP, ISY1, and GAPDH were generated in rabbit against human proteins and were predicted to work in other vertebrates such as chicken. ^2^ GAPDH was used as the housekeeping control gene for comparison in order to determine the target genes’ relative expression. ^3^ The secondary antibody was developed in goat against rabbit IgG and was conjugated to horseradish peroxidase. Secondary-only controls indicated no cross-reactivity.

**Table 3:** Sequence variants identified from the capture array sequencing of 227 kb of GGA 12 from UCD Wingless-2.331 line individuals. Categories of variants were single-nucleotide variants (SNVs) and insertions or deletions (indels). Non-coding variants are those found in an intron, 5000 bases up- or down-stream of a gene, intergenic spaces (greater than 5 kb from a gene), or in a non-coding transcript. Predicted splice region variants are within 1–3 bases of the exon or 3–8 bases of the intron, and exonic variants are found in an exon of a protein coding gene.

227-kb Linked Variants ^1^			
Variant	Non-Coding	Splice Region	Exon
SNVs	503	4	9
Indels	53	0	0

^1^ Linked potentially causative variants were identified by filtering for alignment to the *wg-2* genotype (e.g., a *wg-2/wg-2* mutant must have homozygous alleles for the variant; +/*wg-2* must have heterozygous alleles; and +/+ must be homozygous for the alternate allele).

**Table 4:** Comparison among *wg-2* genotypes of transcript expression for three genes in the linked 227-kb *wg-2* region as determined by RT-qPCR indicates higher *RAF1* transcript expression in mutant and carriers versus wild-type embryos. Three embryos per genotype (*n* = 9 total) were analyzed for each gene and their expression compared to *GAPDH* at Stage 36 HH to determine the relative expression of target gene to housekeeping gene. Note that *RAB43, EFCC1/CCDC48*, and *CECR5*/*HDHD5*, also within the linked region, showed no transcript expression at this stage. Fold change in expression was assessed using the ddCt method [34], and per-plate PCR efficiency (range of 1.52–1.66) was included in each calculation. The *p*-values were calculated using a two-tailed *t*-test with a Bonferroni correction for multiple comparisons [35].

	Fold Changes in Gene Expression					
Gene	*wg-2/wg-2*:+/+	*p*-Value	*wg-2/wg-2*:+/*wg-2*	*p*-Value	+/*wg-2*:+/+	*p*-Value
*RAF1*	1.7 **	0.001	1.2	0.22	1.4 *	0.03
*ISY1*	1.0	1.159	1.1	0.09	0.9	0.11
*CNBP*	1.1	1.383	1.1	1.80	1.1	9.64

* indicates statistically-significant results, using *p* < 0.05 level and ** significant at *p* < 0.001.
